# Advancements in understanding and treating CXCL16/CXCR6 in tumors and in inflammatory diseases: a narrative review

**DOI:** 10.3389/fimmu.2026.1802735

**Published:** 2026-07-20

**Authors:** Xiaohong Li, Shuai Liu, Die Qian, Maokui Huang, Mingmin Na, Qingyan Mo, Mingqian Ju, Jingjing Ge, Jie Gao, Xueling Zhang, Chunping Wan

**Affiliations:** 1 First Clinical Medical College, School of Pharmacy and School of Basic Medicine, Yunnan University of Chinese Medicine, Kunming, China; 2 Department of Scientific Research, The Fifth Affiliated Hospital of Yunnan University of Chinese Medicine, Chuxiong, China

**Keywords:** CXCL16, CXCR6, inflammation, tumor, tumor microenvironment

## Abstract

The CXCL16/CXCR6 axis exerts a multifaceted, bidirectional regulatory influence across various pathological processes, including neoplasms and inflammatory disorders. Its functions are notably context-dependent, possessing the capacity to both inhibit and facilitate disease progression. This article aims to systematically analyze this ‘double-edged sword’ characteristic by providing an exhaustive review of the axis’s functional spectrum across different diseases. Initially, we explore its bidirectional regulatory mechanisms within the tumor microenvironment, emphasizing the influence of tumor heterogeneity and the distinct roles of CXCL16 isoforms (membrane-bound and soluble) in determining functional outcomes. Subsequently, we broaden the discussion to encompass inflammatory diseases, examining the axis’s potential as a predictive marker in these conditions and presenting the latest insights into its role in diseases such as asthma. Additionally, we provide a review of current advancements in drug development targeting this axis and elucidate its mechanisms of action. Ultimately, the paper underscores that accurately identifying the critical nodes where the CXCL16/CXCR6 axis alters its function is the central challenge and opportunity for the development of targeted therapies.

## 1 Introduction

The CXC chemokine family includes CXCL16, which was discovered by Matloubian et al. in 2000 (1). A major member of the CXC chemokine family, consisting of 254 amino acids (2). It is described as a divergent CXC chemokine due to its atypical features among CXC chemokines (3). This formation might lead to distinct structural and functional characteristics. There are three forms present, namely membrane-bound CXCL16 (mCXCL16), soluble CXCL16 (sCXCL16) and CXCL16v (4). mCXCL16 functions as an adhesion molecule, binding to CXCR6-expressing cells and facilitating cell-to-cell interactions. It mediates cell adhesion and the uptake of oxidized lipoproteins. In an inflammatory milieu, cleavage of mCXCL16 by disintegrin and metalloproteinase (ADAM) family proteases releases sCXCL16 (2). sCXCL16 functions as a chemokine, enhancing the migration of immune cells, facilitating the transition from epithelial to mesenchymal states, and promoting the spread of cancer. Research indicates that CXCL16v encodes just the chemokine domain, missing the transmembrane and cytoplasmic parts, and is predominantly found in tissue-resident dendritic cells, although its exact role is still unknown (5). CXCR6 is the sole receptor for sCXCL16, which was previously known as STRL33, ‘Bonzo,’ and TYMSTR (1, 6). It requires binding to sCXCL16 to function and is expressed on activated CD4^+^ and CD8^+^ T cells, natural killer cells (NK), natural killer T cells (NKT), and γ/δ T cells (7). The presence of CXCL16 and CXCR6 indicates an inflammatory environment and associated malignancies, playing a crucial role in regulating inflammation and tumor progression by directly affecting T cell migration and proliferation.

Given that numerous studies did not distinguish between the different forms of CXCL16, the term CXCL16, as used in this context, refers to an unspecified variant that potentially encompasses both forms. When we specifically mention mCXCL16 and sCXCL16, we are addressing the distinct forms of CXCL16. CXCL16/CXCR6 is employed when discussing the two proteins separately, while ‘CXCL16-CXCR6’ denotes the interaction between the proteins upon binding. CXCR6 is a marker of the cytotoxicity and activation level of effector T cells. In addition, the overexpression of CXCL16 is associated with the increased attraction and retention of CXCR6 lymphocytes, and is believed to contribute to the progression of various diseases (5). CXCR6 is primarily expressed on T helper 1 (Th1) or cytotoxic 1 (Tc1) cells and is a marker of immune cell differentiation (5). In immune responses and disease development, the CXCL16/CXCR6 axis has various roles. CXCL16/CXCR6 plays an important role in regulating the homing, activation, amplification, and cytotoxicity of immune cells. Previous reports have mostly focused on immune and inflammatory diseases, such as rheumatic immunology, hepatitis, and the novel coronavirus. In recent years, the immune regulatory role of CXCL16/CXCR6 in malignant tumors exerted through various immune cells (4, 8).

The dual role of the CXCL16/CXCR6 axis in various diseases has yet to be systematically reviewed. This article seeks to fill this gap by providing a comprehensive analysis of the axis’s protective functions in maintaining immune homeostasis and facilitating tissue repair, alongside its detrimental roles in disease progression in pathological conditions such as chronic inflammation and cancer. We conduct a thorough analysis of the molecular and cellular processes that drive its dual actions and evaluate the latest progress in drug development aimed at this pathway. This review aims to furnish researchers in the field with a clear and comprehensive framework of knowledge, thereby offering valuable insights for future basic research and the development of therapeutic strategies.

## 2 Methodology

The literature search was conducted without any temporal restrictions using the PubMed and Web of Science databases. Search terms included “CXCR6,” “CXCL16,” “STRL33,” “Bonzo,” and “TYMSTR,” and Boolean operators (AND/OR) were employed to refine the search results. The search encompassed both *in vitro* and *in vivo* studies involving human and animal subjects, with no limitations regarding preclinical or clinical articles. The selection process was independently executed by the authors, who initially screened titles and abstracts, followed by a comprehensive evaluation of full texts. Any discrepancies in the eligibility assessment were resolved through collaborative discussion among the researchers.

## 3 The CXCL16-CXCR6 axis in tumors exemplifies a quintessential ‘double-edged sword’

The tumor microenvironment, with its complex and immune-regulatory nature, is a key target for cancer therapy. Understanding its features has led to effective treatments, such as immune checkpoint inhibitors and cell therapies (9). These strategies are now being explored beyond cancer, such as using the microenvironment’s immune-suppressive mechanisms to advance psoriasis treatment (10). In recent years, the CXCL16-CXCR6 axis has been widely present in various solid tumors and hematological malignancies, becoming a key hub connecting tumor cells, immune cells, and the tumor microenvironment (TME) (7). A study examining tumor data from the TCGA database determined that genomic alterations such as deletion, amplification, or mutation of CXCR6, which affect its function as the receptor for CXCL16, are infrequent in human cancers. This observation implies that maintaining the integrity and function of this pathway could be essential for the survival or progression of cancer cells (4). The CXCL16-CXCR6 axis in tumors has its effects being highly contingent upon the specific context, significantly influenced by factors such as tumor type, disease stage, and TME. This complexity is further exemplified by the distinct forms of CXCL16: mCXCL16 predominantly facilitates the recruitment and anchoring of lymphocytes to the tumor parenchyma, thereby enhancing anti-tumor immunity. Conversely, sCXCL16 functions as a chemokine that directly interacts with CXCR6-expressing cancer cells, thereby promoting their proliferation, invasion, and metastasis (4, 7). This paper offers an in-depth analysis of current studies on the CXCL16-CXCR6 pathway, elucidating its dual role in tumorigenesis by detailing its functions in both the promotion and inhibition of tumor development.

### 3.1 The CXCL16-CXCR6 axis promotes tumor progression

Numerous tumor cells express the CXCR6 receptor. Upon receiving CXCL16 signals from the surrounding microenvironment, these cells can enhance proliferation, invasion, and metastasis in specific cancer types by activating oncogenic signaling pathways (4). Tumor-associated inflammation serves as a critical early driver of cancer progression by shaping a microenvironment conducive to tumor cell migration and invasion (11). This inflammatory process not only induces the secretion of CXCL16 but also depends on CXCL16 for its maintenance. Consequently, this interaction establishes a self-perpetuating cycle that persistently facilitates the dissemination of tumor cells.

#### 3.1.1 Elevated CXCL16 levels enhance tumor growth

As indicated in the preface, the sCXCL16 is released following the cleavage of mCXCL16 and frequently contributes to the advancement of tumors (12). It has been demonstrated that elevated serum levels of sCXCL16, as measured in ovarian cancer patients, serve as a prognostic indicator of reduced survival, thereby suggesting an increased risk of metastasis. Concurrently, *in vitro* experiments utilizing ADAM10 and ADAM17 inhibitors revealed that the inhibition of CXCL16 shedding from the membrane markedly diminished the migratory capacity of A2780 cells and cultured primary malignant ovarian cancer cells (12). Mir Hina et al. demonstrated that the CXCL16-CXCR6 axis is markedly expressed in non-small cell lung cancer (NSCLC) (13). Notably, the expression levels of CXCR6 were significantly elevated in two specific NSCLC subtypes, adenocarcinoma (AC) and squamous cell carcinoma (SCC), when compared to non-tumorous tissues. Furthermore, CXCR6 expression was more pronounced in AC cells, which exhibited greater migratory and invasive capabilities than SCC cells. These observed differences in migratory and invasive potential between AC and SCC cells were attributed to variations in metalloproteinase expression following CXCL16 stimulation. In the serum of patients with colorectal cancer (CRC), elevated levels of sCXCL16 have been shown to enhance the proliferation, migration, and invasion of CRC cells (14). In gastric cancer (GC), specifically within MKN45 cells, sCXCL16 produced by bone marrow-derived mesenchymal stem cells interacts with the CXCR6 receptor on undifferentiated MKN45 cells, thereby facilitating their proliferation (15). Numerous studies have explored the function of sCXCL16 in prostate cancer, revealing that it promotes the invasion of PC3 and LNCap cells, induces the migration of cancer cells expressing CXCR6, and enhances their proliferation (16–18). Furthermore, both *in vivo* and *in vitro* experiments have demonstrated that sCXCL16 stimulates the proliferation of melanoma IGR37 and IGR39 cells (19). In pancreatic ductal adenocarcinoma (PDAC), increased serum levels of sCXCL16 are associated with heightened invasiveness of PDAC cells. *In vitro* experiments indicate a correlation between sCXCL16 expression and ADAMs (20).

The following studies illustrate instances where the specific phenotype of CXCL16 remains inadequately characterized; however, the CXCL16/CXCR6 axis is implicated in enhancing the regulation, proliferation, and migration of certain tumor cells. CXCL16 and CXCR6, predominantly localized on S-100-positive schwannoma cells, have been shown to augment schwannoma proliferation and facilitate cell migration (21). Additionally, CXCL16 is identified as a direct functional target of miR-451, contributing to the growth and metastasis of osteosarcoma U2OS and SaOS2 cells (22). In PDAC and chronic pancreatitis tissues, CXCL16 and its receptor CXCR6 show increased expression at both mRNA and protein levels compared to normal pancreatic tissues (20). Furthermore, within the prostate cancer cell lines PC-3 and LNCaP, the expression levels of CXCL16-CXCR6 are elevated relative to normal prostate epithelial cells (PrEC), with high CXCL16 expression being correlated with bone metastasis (17). In prostate cancer, elevated expression of CXCL16/CXCR6 is critically involved in tumor growth, proliferation, invasion, and metastasis (23). In comparison to non-tumor tissues, CXCR6/CXCL16 is markedly expressed in NSCLC, with CXCR6 expression being more pronounced in AC than in SCC (13). In GC, CXCL16 expression is higher in tumor tissues compared to adjacent mucosa, whereas CXCR6 exhibits an inverse expression pattern (24). Furthermore, increased CXCR6 expression enhances the invasiveness of hepatocellular carcinoma and contributes to the tumor’s inflammatory microenvironment, correlating with poor patient prognosis (25).

#### 3.1.2 The CXCL16-CXCR6 axis activates various signaling pathways that enhance tumor growth

The CXCL16-CXCR6 axis initiates downstream signaling pathways that collectively contribute to the progression of tumor malignancy. Notably, the Src-FAK-ERK/RhoA pathway functions as a direct mediator of chemotactic migration, swiftly modulating cytoskeletal reorganization to generate the primary force necessary for cell migration (3). This pathway also influences the JAK-STAT3 and PI3K-Akt pathways, thereby regulating the expression of genes associated with proliferation and controlling rapid cell growth and survival (26, 27). Furthermore, the NF-κB pathway serves as a pivotal regulator of the inflammatory microenvironment, facilitating tumor immune evasion by promoting the expression of pro-inflammatory factors and anti-apoptotic proteins (28). Collectively, these pathways constitute a complex, interrelated network that ultimately advances tumor development.

**Figure 1:**
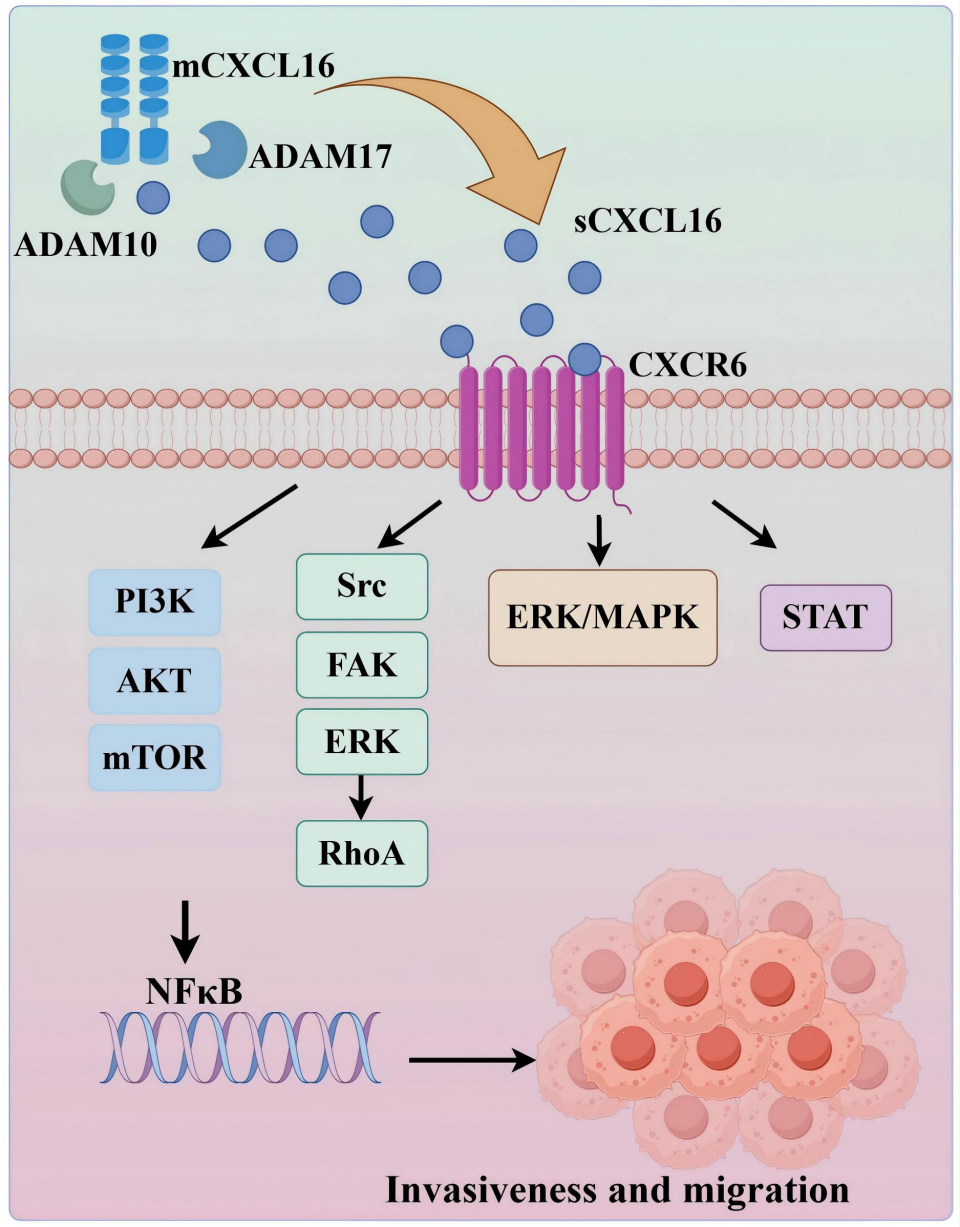
The activation of intracellular signaling pathways is mediated by CXCL16/CXCR6. Within the tumor microenvironment, membrane-bound CXCL16 (mCXCL16) undergoes cleavage by ADAM10/17 proteases, resulting in the formation of soluble CXCL16 (sCXCL16) (As indicated by the yellow arrow). This soluble form of CXCL16 subsequently binds to CXCR6, initiating a cascade of intracellular signaling pathways that play a pivotal role in facilitating cell migration, invasion, and the induction of epithelial-mesenchymal transition (The solid arrow shows verified content).

In breast cancer, the upregulation of ADAM10 leads to an increased release of sCXCL16, which subsequently binds to CXCR6 and activates Src. This activation results in the phosphorylation and activation of FAK, establishing a signaling hub that further stimulates ERK. Moreover, the signaling pathways of FAK and CXCR6 synergistically activate RhoA, which facilitates cytoskeletal reorganization and modulates gene expression. Together, these molecular events mediate the effects of the CXCL16/CXCR6 axis in promoting tumor cell migration and invasion (3). sCXCL16 boosts TNF-α’s cytotoxicity against DLBCL cells (29). In GC, CXCL16 exerts its effects on MKN45 cells in a paracrine manner by activating the STAT3 signaling pathway downstream of its CXCR6 receptor, leading to the direct upregulation of the oncogene Ror1. The CXCL16-STAT3-Ror1 axis constitutes a fundamental mechanism driving the progression of gastric cancer (26). Research on nasopharyngeal carcinoma and neurofibroma suggests that the ERK MAPK signaling pathway is the route through which sCXCL16 facilitates tumor progression (21, 30). In pancreatic ductal adenocarcinoma, PI3K activation not only directly upregulates CXCL16 expression but also enables CXCL16 to further activate PI3K, establishing a CXCL16/PI3K autocrine positive feedback loop that promotes tumor growth (27). In prostate cancer, the migration of PC3 and C4-2B cells is facilitated through the activation of the CXCR6 receptor, which subsequently engages the downstream PI3K, Akt/PKB, and mTOR signaling pathway (31). Furthermore, the PI3K-Akt pathway is integral to the migration processes of SKOV-3 (ovarian cancer) and BHP10-3 (thyroid cancer) cells (28, 32). In the context of ovarian cancer, tumor-associated macrophages (TAMs) enhance the CXCL16-CXCR6 axis via the NFκB and PI3K/Akt pathways, thereby promoting tumor invasion and migration (28) (see **Figure 1**).

#### 3.1.3 The CXCL16-CXCR6 axis attracts cells to form an immunosuppressive environment

As a central immune regulatory mechanism, the CXCL16/CXCR6 axis is expressed in tumor cells and extensively in various cells of the tumor microenvironment. This diverse expression facilitates the concurrent recruitment of lymphocytes with anti-tumor capabilities and myeloid cells that exert immunosuppressive effects, thereby creating a dynamic competitive interaction between these cell types. Nevertheless, in the later stages of tumor progression, the pro-tumorigenic effects of this axis tend to predominate, underscoring its dual role (3).

The interaction between CXCL16-CXCR6 and MSCs, tumor-associated TAMs, and MDSCs is implicated in immunosuppression and the facilitation of tumor progression (3). As a key source of CXCL16, cancer-associated fibroblasts (CAFs) are vital to the activity of oncogenic fibroblasts, emitting a range of substances that support cancer advancement. This activity is particularly significant in the context of triple-negative breast cancer and NSCLC (33, 34). Upon recruitment to the tumor microenvironment, MSCs exhibit elevated expression of CXCL16 in response to local stimuli. Consequently, CXCL16 attracts a substantial influx of immunosuppressive cells, such as regulatory T cells, and collaborates with MSCs to significantly suppress antitumor immunity, thereby facilitating immune evasion. In GC, the Wnt5a-Ror2 signaling pathway is vital in bone marrow-derived mesenchymal stem cells, resulting in the production of sCXCL16, which then enhances cancer cell growth (15). The CXCL16/CXCR6 axis is also present in prostate and breast cancers, where it stimulates MSCs to enhance the metastatic progression of cancer cells. Furthermore, in breast cancer, cytokines produced by mesenchymal stem cells facilitate the recruitment of tumor-associated macrophages and myeloid-derived suppressor cells (35, 36). Macrophages are pivotal in establishing the tumor inflammatory environment, and sCXCL16 may function as a chemokine that promotes macrophage polarization. The feedback interaction between the CXCL16/CXCR6 axis and macrophages is particularly pronounced in the maintenance and progression of gliomas, as microglia, the resident macrophages of the brain, are key drivers of glioma progression and can express CXCL16/CXCR6 (37). Additionally, the CXCL16/CXCR6 pathway is adjusted by macrophages to support the migration and invasion of glioma (38).

The CXCL16/CXCR6 axis does not directly initiate the epithelial-mesenchymal transition (EMT) program; however, it indirectly and significantly facilitates this process by recruiting specific immune cells and activating key signaling pathways (3). While the role of chemokines in EMT regulation is well documented, evidence specifically implicating CXCL16 in this context remains relatively sparse. Importantly, ADAM10-mediated proteolytic shedding of CXCL16 has been identified as a crucial step in promoting EMT. Research in GC has demonstrated that CXCL16 induces EMT by activating the Akt and MAPK signaling pathways, which is characterized by the upregulation of key EMT-related transcription factors such as Snail, Slug, and β-catenin, alongside the downregulation of the epithelial marker E-cadherin (39). Further investigations suggest that once cancer cells acquire an EMT phenotype and form subpopulations with enhanced invasiveness and migratory capacity, their infiltration into specific “gateway” sites around blood vessels is contingent upon mechanochemical chemotaxis guided by chemokines (40).

### 3.2 The CXCL16-CXCR6 axis inhibits tumor progression

#### 3.2.1 CXCL16/CXCR6 axis mediates immune cell recruitment

CXCL16, functioning as a chemokine, interacts with the CXCR6 receptor located on the surface of lymphocytes, thereby initiating intracellular signaling pathways associated with activation (3). This interaction significantly augments the activity and functionality of these effector lymphocytes. Upon activation, cytotoxic T cells and NK cells secrete perforin, granzymes, and other cytotoxic substances, which facilitate the formation of pores in the tumor cell membrane and induce apoptosis (4). Concurrently, this process promotes Th1-type immune responses, thereby enhancing immune surveillance (41). Additionally, activated lymphocytes exhibit elevated expression of Fas ligand and other molecules, further facilitating the programmed cell death of tumor cells via the death receptor pathway (42). Within the tumor microenvironment, dendritic cells (DCs) secrete CXCL16, thereby establishing a localized chemokine milieu around blood vessels. This milieu facilitates the selective recruitment of CXCR6-positive immune cell subsets, including cytotoxic CD8^+^ T cells, tissue-resident memory T cells (TRM), NKT cells, and intraepithelial lymphocytes (IELs), all of which exhibit potent anti-tumor activity. These CXCR6-positive subsets help inhibit the growth and spread of tumor cells. The CXCL16-CXCR6 axis supports sustained interactions between cytotoxic T lymphocytes (CTLs) and DCs within the perivascular tumor stroma, thereby optimizing anti-tumor efficacy (3, 43). In the tumor context, CXCR6-expressing T cells are recruited by CXCL16 and become activated upon encountering CXCR6-expressing DCs. This activation leads to their expansion, localization to the tumor site, and execution of tumoricidal functions. While the role of CXCL16 in tumors remains somewhat uncertain, CXCR6-expressing CD8^+^ T cells are pivotal in mediating anti-tumor responses. Research conducted by Di Pilato (8) and Wang, B (44) underscores the critical importance of CXCR6 in CTL-mediated tumor control.

#### 3.2.2 CXCR6^+^CD8^+^ T cells are highly cytotoxic

CXCR6 T cells are recognized for their high pathogenic potential. Previous studies and review articles have explored the beneficial role of CXCR6^+^CD8^+^ T cells in tumor contexts (8, 45). This subset of CD8^+^ T cells is characterized by enhanced cytotoxic capabilities. In immunologically active tumors, CXCR6^+^CD8^+^ T cells demonstrate the ability to effectively identify and release cytotoxic agents, such as perforin (PRF) and granzymes (GMZ), to directly target and eradicate cancer cells. Additionally, as a crucial subset of effector memory T cells, they possess the capacity for long-term persistence within tumors, thereby sustaining their functional activity and contributing to continuous immune surveillance. Research has demonstrated that both murine and human breast cancer cells secrete the active chemokine CXCL16, which can interact with the CXCR6 receptor on Th1 cells, thereby activating CD8 effector T cells and facilitating their recruitment to inflammatory sites. Moreover, CXCL16 enhances the movement of activated NK cells with CXCR6 towards breast cancer cells that have been irradiated, boosting the anti-tumor effectiveness of NK cells (46, 47). In the context of epithelial ovarian cancer, CXCR6 expression is significantly correlated with lymph node metastasis, suggesting that the CXCL16/CXCR6 axis may play a critical role in tumor growth, proliferation, invasion, and metastatic potential (48). Furthermore, elevated CXCL16 expression in colorectal cancer tumors serves as a positive prognostic indicator and is associated with increased levels of tumor-infiltrating lymphocytes (49). Both human and murine studies highlight that CXCR6 is significantly present on TRM cells, essential for their localization in tumor tissues and improving protective responses against ovarian cancer (50). In muscle-invasive bladder cancer (MIBC), CXCR6 is predominantly expressed in T cells and NK cells, facilitating T/NK-myeloid interactions via the CXCL16-CXCR6 axis. Notably, CXCL16-expressing macrophages and dendritic cells recruit CXCR6^+^ T and NK cells, which exhibit enhanced cytotoxicity, thereby augmenting anti-tumor immunity (51). The extent of intratumoral infiltration by CXCR6^+^CD8^+^ T cells is a crucial determinant of prognosis in patients with SCLC. Consequently, strategies to increase the number of intratumoral activated DCs or directly enhance the infiltration of CXCR6^+^CD8^+^ T cells may improve therapeutic outcomes in SCLC (52). Through the CXCL16/CXCR6 axis, LGMN^+^ macrophages could draw ICEP2^+^ CD8^+^ T cells into the tumor core, which may contribute to resistance against anti-PD-1 therapy in GC (53, 54).

#### 3.2.3 The multifaceted role of CXCL16 in tumorigenesis

In contrast to the role of sCXCL16 in promoting tumor progression in the majority of cancers, Hua Yang et al., through *in vitro* investigations on DLBCL, demonstrated that sCXCL16 activates the NF-κB signaling pathway via binding to CXCR6. This activation leads to an increase in extracellular TNF-α concentration, upregulation of ADAM10 expression to induce sCXCL16 secretion, enhancement of TNF-α-induced apoptosis, and inhibition of tumor cell proliferation (29). Furthermore, analyses of renal cancer patient biopsies revealed that CXCL16 expression is associated with reduced growth of renal cell carcinoma (RCC), with patients exhibiting high CXCL16 expression showing significantly improved survival rates compared to those with low expression (55). The aforementioned studies indicate that the specific function of sCXCL16 in tumors may be contingent upon the tumor type, disease stage, and underlying mechanism of action. Furthermore, research conducted by Hald et al. (34) demonstrated through clinicopathological observations of NSCLC that elevated expression of mCXCL16 in stromal-associated tumors correlates with increased survival rates. However, studies by Hu et al. 56) and Mir et al. (13) suggest that sCXCL16 augments the invasiveness of NSCLC cells, implying that CXCL16 may assume distinct roles in lung cancer contingent upon its soluble or cellular form. Details are provided in **Table 1**.

**Table 1:** The impact patterns of various CXCL16 isoforms on different tumor types.

Tumor type	Species	CXCL16 type	Pro-tumor or anti-tumor	Sample type	Ref.
Ovarian Cancer	Human	sCXCL16	Pro-tumor	Serum	(12)
Lung Cancer	Human	sCXCL16	Pro-tumor	Serum	(13)
Lung Cancer	*In vitro* cell experiment	Not explained	Pro-tumor	Cell Viability	(56)
Colorectal Cancer	Human	sCXCL16	Pro-tumor	Serum	(14)
Gastric Cancer	*In vitro* cell experiment	sCXCL16	Pro-tumor	Culture Supernatants	(15)
Prostate Cancer	*In vitro* cell experiment	sCXCL16	Pro-tumor	Culture Supernatants	(18)
Prostate Cancer	Human	Not explained	Pro-tumor	Tumor Tissue	(17)
Pancreatic Ductal Adenocarcinoma	Human	mCXCL16 sCXCL16	Pro-tumor	Pancreatitis Tissues and Serum	(20)
Schwannomas	Human	mCXCL16	Pro-tumor	Tumor Tissue	(21)
Diffuse Large B-cell Lymphoma	*In vitro* cell experiment	mCXCL16	Anti-tumor	Annexin V Staining	(29)
Non-Small Cell Lung Cancer	Human	mCXCL16	Anti-tumor	Tumor Tissue	(34)
Renal Cell Cancer	Human	mCXCL16	Anti-tumor	Tumor Tissue	(55)

**Figure 2:**
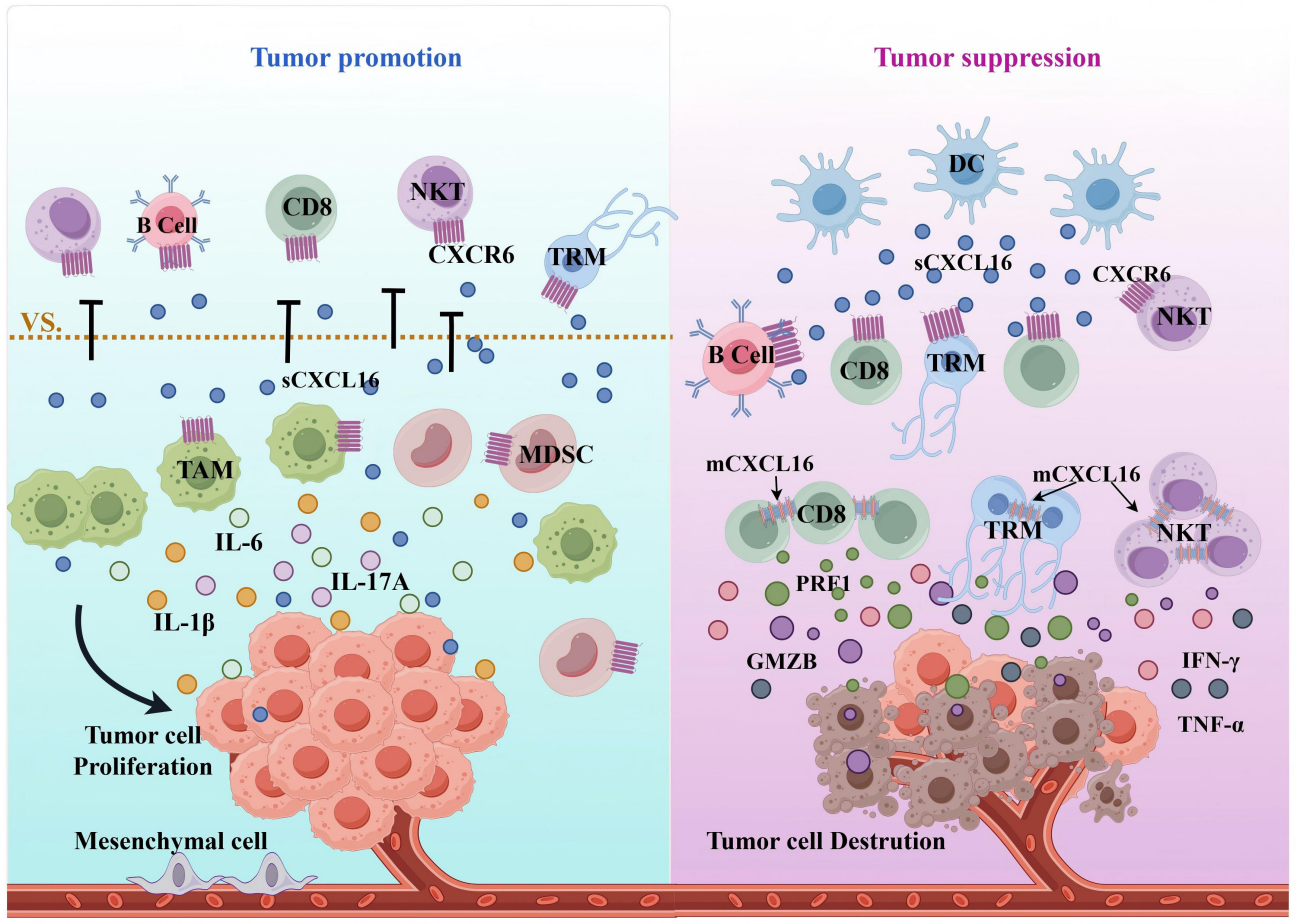
Mechanistic diagram of the dual role of CXCR6-CXCL16 in tumor promotion and tumor suppression. Tumor Promotion (Left Diagram): The anti-tumor T cell subsets encompass CXCR6^+^CD8^+^ T cells, tissue-resident memory T cells (TRM), natural killer T cells (NKT), and B cells, as illustrated in the top left schematic. Soluble CXCL16 (sCXCL16), secreted by tumor cells and the tumor microenvironment, chemotactically attracts CXCR6^+^ mesenchymal stem cells (MSCs), tumor-associated macrophages (TAMs), and myeloid-derived suppressor cells (MDSCs) to the tumor site. This recruitment establishes local immunosuppression and induces exhaustion of the aforementioned anti-tumor T cell subsets, as depicted in the center of the diagram. Concurrently, inflammatory factors released by TAMs and other cells, such as IL-6 and TNF-α, further facilitate tumor progression, as shown in the bottom left diagram. Tumor Suppression (Right Diagram): sCXCL16, secreted by dendritic cells (DCs), plays a pivotal role in recruiting CXCR6^+^CD8^+^ T cells, NKT cells, and other lymphocyte populations, including additional DCs, into the tumor microenvironment (refer to the top right schematic). The mCXCL16 further enhances the recruitment of these anti-tumor T cell subsets, facilitating the release of perforin, granzymes, and other effector molecules, thereby intensifying the immune-mediated pressure against tumor cells (refer to the bottom right schematic). Collectively, the CXCL16-CXCR6 signaling axis exerts a dual regulatory function within the tumor microenvironment, simultaneously promoting tumor progression through the recruitment of immunosuppressive cells and inhibiting tumor growth by attracting effector T cells.

In conclusion, the primary mechanism through which the CXCL16/CXCR6 axis impedes tumor proliferation involves the strategic recruitment and activation of specific immune cells, particularly lymphocytes, to indirectly exert a potent anti-tumor effect. Details are provided in **Table 2**. This axis enhances the homing capability of lymphocytes to tumor sites and optimizes their cytotoxic potential, ultimately facilitating the effective eradication of tumor cells and thereby inhibiting tumor growth. The capacity to induce targeted immune cell infiltration underscores its potential as a therapeutic target for anti-tumor strategies (see **Figure 2**).

**Table 2:** The favorable prognostic impact of CXCR6^+^ immune cells in the tumor microenvironment.

Tumor type	Species	T cell type	Favorable prognosis	Ref.
Ovarian Cancer	Human and Mouse	CXCR6^+^CD8^+^Trm cell	up	(50)
Triple-negative breast cancer	Human	CXCR6^+^CD8^+^	up	(57)
Colon Cancer	Mouse	CXCR6^+^CD8^+^	up	(44)
Colon Cancer	Human	CXCR6^+^TAMs	down	(58)
Bladder cancer	Human	CXCR6^+^CD8^+^ CXCR6^+^NK Cell	up	(51)
Small cell lung cancer	Mouse	CXCR6^+^CD8^+^DC	up	(52)
Gastric cancer	Mouse Human	ICEP2 CD8^+^ CXCR6^+^CD8^+^	up	(53, 54)
Metastatic melanoma	Mouse	CXCR6^+^CD8^+^	up	(59)
Hepatocellular carcinoma	Human Mouse	CXCR6^+^CD8^+^	up	(60, 61)
Nasopharyngeal carcinoma	Human	CXCR6^+^CD8^+^	up	(62)

## 4 Inflammatory diseases

**Figure 3:**
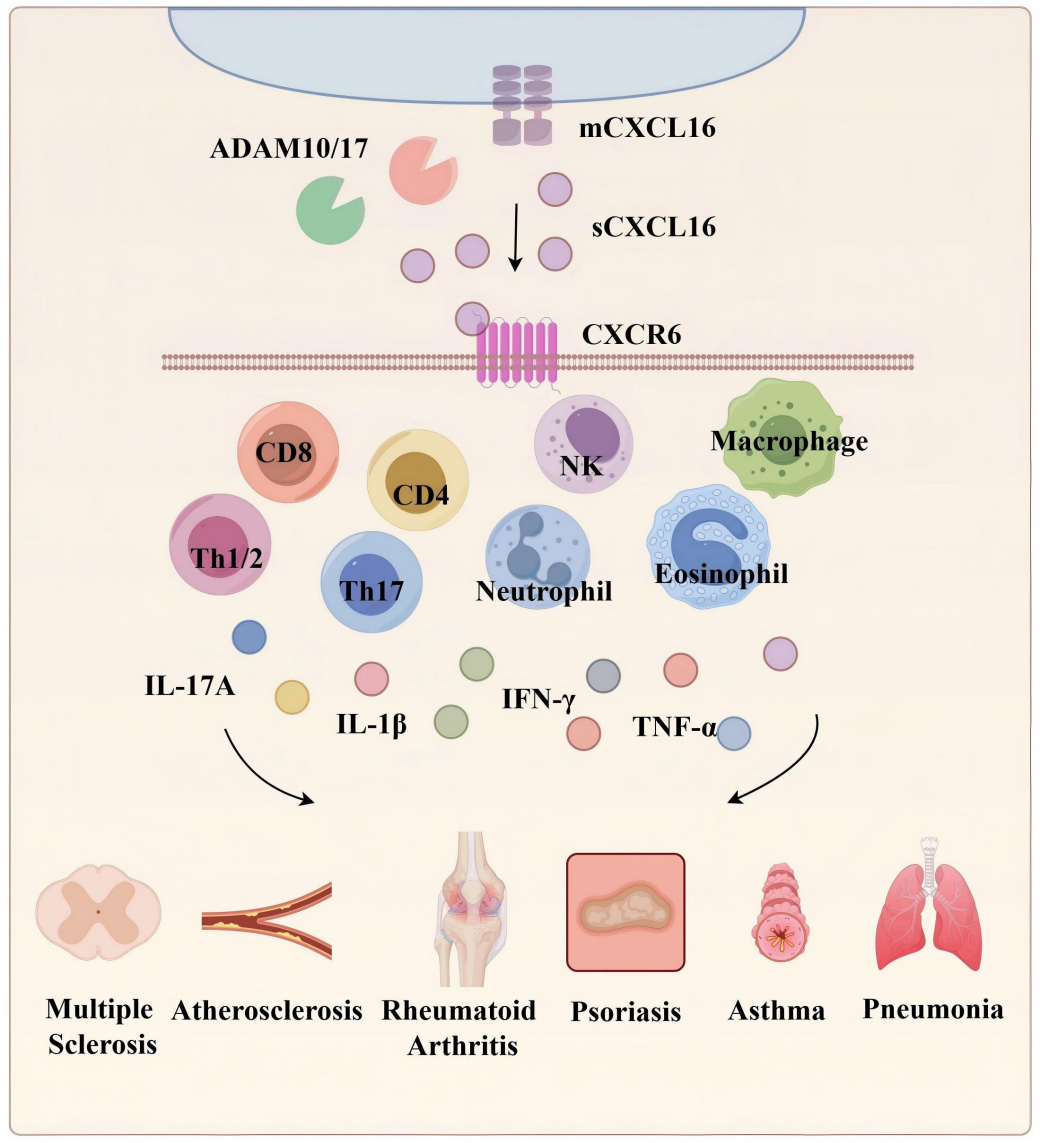
The CXCL16/CXCR6 axis recruits immune cells to promote the progression of inflammatory diseases. Upon cleavage of membrane-bound CXCL16 (mCXCL16) by the proteases ADAM10 and ADAM17, soluble CXCL16 (sCXCL16) is released. Persistent inflammatory stimuli can result in increased levels of sCXCL16. This soluble chemokine facilitates the recruitment of CXCR6-expressing pathogenic T cells—including Th1/2, Th17 cells, neutrophils, eosinophils, cytotoxic T cells, and macrophages—to sites of lesions via a CXCR6 receptor-dependent mechanism. Activation of these recruited CXCR6-positive pathogenic T cells leads to the production of significant quantities of key pathogenic cytokines, such as interleukin-17A (IL-17A), interferon-gamma (IFN-γ), and tumor necrosis factor-alpha (TNF-α), thereby exacerbating the progression of diseases such as multiple sclerosis, atherosclerosis, rheumatoid arthritis, psoriasis, pneumonia and asthma.

In the context of inflammatory diseases, mCXCL16 primarily facilitates the local recruitment and retention of immune cells, while sCXCL16 is involved in the chemotaxis of inflammatory cells over longer distances. These two forms collaborate to initiate and sustain an inflammatory response (63). Nonetheless, the mechanisms by which CXCR6 operates in various immune cells, such as Th17 and NKT cells, exhibit significant differences. Current research predominantly concentrates on individual cell types, and there remains a paucity of systematic analyses concerning CXCR6-mediated intercellular interactions and synergistic effects. Furthermore, the associated molecular pathways require further elucidation (63, 64). Consequently, modulating the balance of CXCL16 expression or directly targeting CXCR6 to inhibit pathological cell recruitment could potentially redirect this axis towards beneficial regulation, thereby offering novel therapeutic strategies for inflammation-related diseases (4) (see **Figure 3**).

### 4.1 The CXCL16/CXCR6 axis may serve as a biomarker for predicting and assessing inflammatory disease risk and severity

#### 4.1.1 Multiple sclerosis

The primary pathogenic mechanism underlying multiple sclerosis (MS) involves a CD4^+^ T cell-mediated autoimmune response (65). These T cells compromise the integrity of the blood-brain barrier, resulting in demyelination within the central nervous system and subsequent progressive neurological dysfunction. Investigations utilizing experimental autoimmune encephalomyelitis (EAE), an established animal model of MS, have demonstrated that CXCR6 is markedly expressed in a subset of pathogenic CD4^+^ T cells characterized by rapid proliferation, differentiation, and the secretion of substantial quantities of inflammatory cytokines (66). These CXCR6^+^ CD4^+^ T cells produce various inflammatory mediators, including interleukin -17 (IL-17), Granulocyte-Macrophage Colony Stimulating Factor (GM-CSF), TNF-α, and interferon-gamma (IFN-γ), which directly contribute to myelin degradation and neuronal damage (67). Evidence suggests that targeting and depleting CXCR6^+^CD4^+^ T cells infiltrating the spinal cord with anti-CXCR6 antibodies not only effectively prevents the onset of EAE in murine models but also exerts significant therapeutic effects in mice that have already manifested the disease or paralysis, thereby reversing disease progression and ameliorating neurological symptoms (68).

#### 4.1.2 Atherosclerosis

In the context of atherosclerosis, sCXCL16 is significantly upregulated in vascular endothelial cells upon activation by inflammatory stimuli, playing a crucial role in the disease’s pathophysiology. The proteolytic cleavage of mCXCL16 by ADAM10/17 proteases results in the release of sCXCL16 into the bloodstream, with sustained inflammatory stimulation causing elevated plasma levels of sCXCL16. Through a mechanism dependent on the CXCR6 receptor, sCXCL16 facilitates the initial rolling and capture of platelets on the vessel wall. Concurrently, mCXCL16, anchored on the cell membrane, functions as a potent adhesion ligand, providing a stable attachment point for platelets. This process ultimately leads to abnormal platelet aggregation on the plaque surface, thereby increasing the risk of thrombosis (69, 70). Furthermore, as a chemokine, CXCL16 effectively recruits inflammatory cells, including Th1 cells, CD8^+^ T cells, and NK cells, to the vascular intima via its specific receptor CXCR6, thereby directly exacerbating the inflammatory response (71). Research has demonstrated that soluble CXCL16 serves as a potential biomarker for atherosclerosis, thereby establishing a connection between inflammation and metabolic risk. Its pro-inflammatory effects are notably intensified in the context of acute coronary syndrome (72). Genetic evidence suggests that certain CXCL16 haplotypes may exacerbate plaque formation by upregulating its signaling pathways, including the activation of P2X7R (73, 74). At lesion sites, cytokines such as IFN-γ, IL-2, and IL-15 collectively modulate the CXCL16/CXCR6 axis, thereby advancing disease progression through the facilitation of foam cell formation and T cell homing (75). Current research suggests that sCXCL16 may have favorable biomarker characteristics for epidemiological and clinical applications, and some scholars have proposed that targeting and inhibiting CXCL16 could be a promising approach for AS treatment (72, 76).

#### 4.1.3 Rheumatoid arthritis

The CXCL16/CXCR6 axis plays a crucial pro-inflammatory role in the pathogenesis of rheumatoid arthritis (RA) (77). Its primary function is to facilitate the targeted migration of pathogenic immune cells to joint sites. In response to inflammatory mediators such as TNF-α and IL-17, fibroblasts and endothelial cells within the synovium exhibit elevated expression of the chemokine CXCL16. Concurrently, activated pathogenic immune cells in the peripheral circulation, notably Th17 cells and cytotoxic CD8^+^ T cells, express high levels of their exclusive receptor, CXCR6. As these CXCR6-expressing cells traverse inflamed joints, they are selectively recruited, adhere to, and infiltrate the synovial tissue, guided by the chemotactic gradient established by CXCL16. Upon infiltrating the joint, Th17 cells secrete substantial quantities of potent pro-inflammatory mediators, such as IL-17, which further activate synovial cells, thereby exacerbating inflammatory responses and promoting osteoclast differentiation, leading to bone erosion (78). Critically, this process establishes a self-amplifying positive feedback loop: the factors secreted by infiltrating cells stimulate increased local production of CXCL16, which subsequently recruits additional inflammatory cells. This recruitment perpetuates chronic inflammation and contributes to the formation of pannus and joint destruction. Clinical studies have demonstrated that sCXCL16 levels in the serum of patients with RA are significantly elevated and positively correlate with disease activity (79).

In-depth research has demonstrated that CXCR6-positive T cells within the synovium exhibit heightened activation. Cytokines, notably IL-15, can stimulate T cells to upregulate CXCR6, thereby enhancing their migration towards CXCL16 and promoting the secretion of IFN-γ (77). In the context of juvenile idiopathic arthritis, this axis collaborates with CXCR3 to facilitate T cell recruitment (80). Additionally, the CXCL16-CXCR6 axis directly contributes to bone destruction by upregulating RANKL expression through the activation of signaling pathways such as JAK2/STAT3, consequently promoting osteoclast differentiation (81, 82). Importantly, in certain pathological scenarios, such as prosthetic joint infections, this axis may alternatively recruit CXCR6-positive regulatory T cells to establish an immunosuppressive microenvironment, potentially creating a deleterious feedback loop, thereby indicating its context-dependent functionality (83). Consequently, targeting the CXCL16-CXCR6 axis presents a promising strategy for precise intervention in arthritis-related inflammation, bone destruction, and pathological immune microenvironments.

#### 4.1.4 Psoriasis

The CXCL16-CXCR6 signaling pathway constitutes a fundamental mechanism underlying the inflammatory processes in psoriasis (84, 85). Upon stimulation by cytokines such as IL-17 and TNF-α, keratinocytes, dendritic cells, and other skin-resident cells exhibit elevated expression of CXCL16. This chemokine specifically attracts CXCR6-expressing pathogenic T helper 1 (Th1), Th17, cytotoxic T lymphocytes, and neutrophils from the circulatory system to the dermal environment (84). Once recruited, these immune cells become activated and secrete substantial quantities of critical inflammatory mediators, including IL-17A and IFN-γ. These mediators directly induce hyperproliferation of keratinocytes and epidermal thickening, thereby establishing a self-perpetuating positive feedback loop (86). In this loop, inflammatory cytokines further stimulate cutaneous cells to produce additional CXCL16, perpetually attracting inflammatory cells and contributing to the persistence of chronic lesions (87). This axis also plays a key role in disease relapse. Pathogenic tissue-resident memory T cells exhibit high expression of CXCR6, facilitating their prolonged retention in the skin (88). Upon activation, these cells not only secrete inflammatory mediators but also stimulate keratinocytes to produce additional chemokines, thereby establishing a complex inflammatory network that perpetuates chronic relapse (88). Research indicates that UBE2L3 deficiency, along with upstream signaling pathways such as TLR7, contributes to this process by upregulating CXCL16 (85, 89). Consequently, the CXCL16/CXCR6 axis is identified as a pivotal component in the psoriasis inflammatory circuit and represents a promising therapeutic target. Inhibiting this pathway may enable the precise attenuation of skin-specific inflammation.

#### 4.1.5 Pneumonia

The CXCL16/CXCR6 axis serves a pivotal dual function in pulmonary infections. Upon pathogen invasion, lung tissue cells upregulate CXCL16, which chemotactically attracts CXCR6-expressing NKT cells, ILCs, and CD8^+^ T cells to the site of infection (90). However, excessive activation of these recruited immune cells can precipitate a “cytokine storm” and cause damage to alveolar epithelial cells, culminating in acute lung injury or acute respiratory distress syndrome (ARDS) (91). This phenomenon is particularly evident in severe cases of influenza and COVID-19, where elevated serum levels of sCXCL16 in patients are associated with increased disease severity (92). Additionally, this axis is a crucial regulator of effector T cell localization in granulomatous inflammation (93, 94). Moreover, CXCL16 can directly exacerbate lung injury through mechanisms such as the induction of reactive oxygen species (ROS) and disruption of the epithelial barrier (95). Therefore, this axis is one of the core drivers of pulmonary inflammation and damage.

#### 4.1.6 Asthma

In the airways of individuals with asthma, airway epithelial cells, smooth muscle cells, and other cellular components exhibit elevated expression of CXCL16 upon stimulation by allergens or cytokines, such as IL-4 and IL-13 (96). This expression facilitates the chemotactic attraction of CXCR6-expressing Th2 cells to the airway mucosa. Th2 cells play a pivotal role in type 2 inflammation associated with asthma, as they secrete IL-4, IL-5, and IL-13, thereby enhancing eosinophil infiltration, immunoglobulin E (IgE) production, and mucus secretion. The IL-13 produced by the recruited Th2 cells can further induce the expression of CXCL16 in airway structural cells, creating a positive feedback loop that amplifies and perpetuates the inflammatory response (97). Zou and colleagues administered anti-CXCR6 antibodies to OVA-sensitized asthmatic mice, resulting in the effective depletion of CXCR6-expressing CD4 T cells in the pulmonary tissue of these mice. This treatment led to a marked reduction in the protein levels of CXCR6 and CXCL16, which, in turn, significantly mitigated airway inflammation, mucus hypersecretion, serum anti-OVA IgE concentrations, and the levels of IL-17A and IFN-γ, as well as inflammatory cell infiltration in lung tissue (98). In parallel, Liu and colleagues conducted a study in which CXCL16 was knocked out in mice, demonstrating a downregulation of H2-DM. This downregulation impaired dendritic cell antigen processing and presentation, thereby attenuating Th2-mediated airway inflammation and reducing peribronchial inflammatory cell infiltration, airway mucus secretion, and the expression of mucus-associated proteins (99). Zou et al.*’s* investigation into the CXCL16/CXCR6 axis in asthma aligns with the findings of Liu et al. (99). Targeting the CXCR6-CXCL16 axis emerges as a promising novel strategy for the treatment of asthma, particularly in Th2-dominant and high-eosinophilic phenotype asthma.

### 4.2 The CXCL16-CXCR6 axis plays a positive role in atherosclerosis. mCXCL16 acts as a scavenger receptor

**Figure 4:**
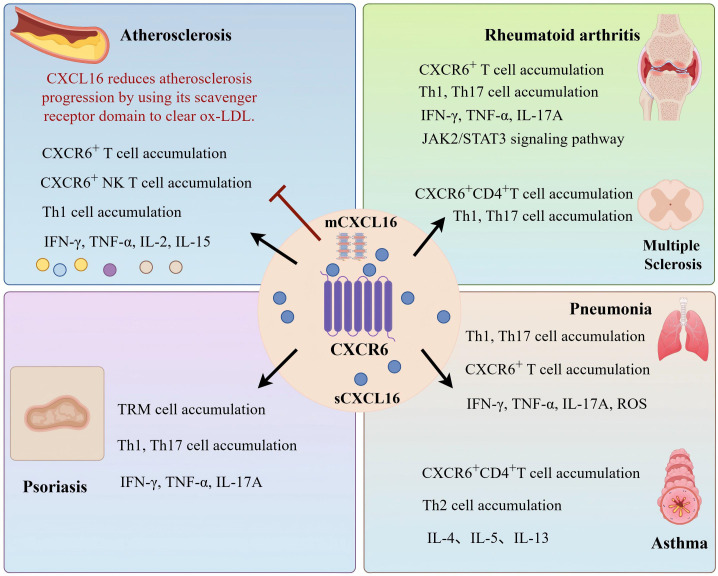
The function of the CXCL16/CXCR6 axis in inflammatory diseases. CXCL16 exhibits a dual role in the pathogenesis of atherosclerosis. On the one hand, CXCL16 may confers a protective effect through its scavenger receptor domain (SR-PSOX) (The red text and arrows require further research and confirmation). Conversely, CXCL16 interacts with its receptor, CXCR6, facilitating the recruitment of activated T cells and natural killer (NK) cells to atherosclerotic lesions. This interaction exacerbates local inflammatory responses and contributes to plaque progression and instability, manifesting pathogenic effects. The CXCL16/CXCR6 axis is instrumental in directing the migration of immune cells to sites of inflammation in various diseases, including rheumatoid arthritis, psoriasis, asthma, pneumonias, and multiple sclerosis. In these conditions, it promotes the release of pro-inflammatory mediators (The solid arrow shows verified content).

In the initial phases of atherosclerosis, dysfunction of endothelial cells facilitates the infiltration and retention of low-density lipoprotein (LDL) within the subendothelial space, where it undergoes oxidized low-density lipoprotein (ox-LDL). Oxidized LDL catalyzes chronic inflammatory responses and acts as a primary factor in the development of atherosclerotic plaques (100). During this period, the mCXCL16 present on activated vascular smooth muscle cells, macrophages, and other cell types specifically recognizes, binds, and internalizes ox-LDL, as well as cellular debris from apoptotic or necrotic cells, via its scavenger receptor domain (74). This clearance mechanism effectively reduces the accumulation of ox-LDL within the vessel wall and prevents apoptotic cells from advancing to secondary necrosis. Consequently, in the early stages of atherosclerosis, the proper functioning of mCXCL16 is crucial for maintaining vascular wall homeostasis and delaying plaque progression. Nonetheless, when lipid accumulation or inflammatory stimuli persist, this protective mechanism can be compromised. Macrophages that internalize substantial quantities of ox-LDL can differentiate into foam cells. When the burden of these lipids surpasses the macrophages’ clearance capacity, it may lead to apoptosis and necrosis, thereby aggravating plaque instability. This indicates that during the initial stages of atherosclerosis (AS), the moderate scavenging function of mCXCL16 serves a protective role. In contrast, during the intermediate to advanced stages of AS, excessive activation of mCXCL16 may contribute to the disease’s progression (71, 101) (see **Figure 4**).

## 5 Pharmacological agents targeting the CXCL16/CXCR6 axis

Through a systematic review of the literature, it was found that drugs primarily acting on the CXCL16/CXCR6 axis include natural compounds, receptor inhibitors, and statins. Based on the different forms of CXCL16 on this axis mediating distinct biological functions, the mechanisms of action are mainly divided into two parts. On one hand, these drugs directly act on CXCR6, inhibiting the expression of CXCR6 and preventing the recruitment of CXCR6-positive immune cells (88, 102–106); on the other hand, by interfering with the activation of ADAM 10, they inhibit the cleavage of mCXCL16, increase the accumulation of functional mCXCL16 on the membrane, and further reduce the generation and release of sCXCL16 (107–109). Details are provided in **Table 3**.

**Table 3:** Summary of pharmacological preparations targeting the CXCL16/CXCR6 axis.

Drug name	Diseases	Mechanism of action.	Development stage	Ref.
Baicalein	Multiple Sclerosis	Limit the infiltration of CXCR6^+^CD4^+^ and CXCR6^+^CD8^+^ T cells in the central nervous system and specifically target pathogenic CXCR6^+^Th17 cells.	Preclinical research	(102)
Curcumol	Psoriasis	Block the CXCL16-CXCR6 interaction by downregulating CXCR6 to inhibit TRM cell activity and inflammation.	Preclinical research	(88)
Statins	Coronary artery disease	Inhibit protease A disintegrin and metalloproteinase 10 (ADAM10) to downregulate CXCL16 release	Clinical trials	(107)
P2X7R inhibitor A438079	Kidneys of children with primary nephrotic syndrome	Reduce CXCL16 release	Preclinical research	(103)
Alirocumab	Familial hypercholesterolemia	Inhibit PCSK9 to reduce the CXCR6^+^cells and CXCL16 release	Clinical trials	(104)
Compound 81 and Compound 17	Hepatocellular carcinoma	Reduce the expression of CXCR6	Preclinical research	(110)
Resveratrol	Type 1 Diabetes Mellitus in Mice	Inhibit the ADAM10-mediated proteolytic cleavage of CXCL16 and subsequently impede the CXCL16-facilitated recruitment of T cells to pancreatic β cells.	Preclinical research	(108)
GI254023X	Not provided	Inhibit ADAM10 to reduce the release of sCXCL16	Preclinical research	(109)
Escin	AGS human gastric cancer cells	Reduces the expression of sCXCL16 while increasing the expression of its transmembrane form, without affecting the expression of the CXCR6 receptor.	Preclinical research	(105)
SBI-457	Hepatocellular Carcinoma	Antagonize CXCR6 and disrupt CXCR6/β-catenin crosstalk	Preclinical research	(106)
MAPKK inhibitor	Atherosclerosis	Reduce CXCL16 release	Preclinical research	(111)
Anti-CXCL16 neutralizing antibodies	*Salmonella enterica* serovar Enteritidis infection Crescentic Glomerulonephritis	Block CXCL16 activity	Preclinical research Preclinical research	(112) (113)

## 6 Summary and outlook

The CXCL16/CXCR6 axis plays a dual role in tumor immunity, primarily due to the distinct, opposing biological functions mediated by different forms of CXCL16. On one hand, this axis serves as a critical regulator of anti-tumor immunity; elevated expression of CXCR6 within the tumor microenvironment facilitates CD8^+^ T cell infiltration and correlates with improved outcomes of anti-PD-1 therapy, indicating its potential as a target for enhancing tumor immunotherapy (44, 114). Conversely, the sCXCL16, produced through ADAM10-mediated cleavage of mCXCL16, may contribute to tumor progression by fostering an immunosuppressive microenvironment, mediated by TAMs or MDSCs, thereby impairing T cell function. Consequently, intervention strategies targeting this axis must be highly precise: it is imperative to both enrich or activate CXCR6^+^ T cells to bolster anti-tumor immunity and inhibit ADAM10-mediated cleavage of CXCL16 to mitigate the pro-tumor effects of sCXCL16, thereby optimizing therapeutic efficacy through bidirectional regulation.

While contemporary research predominantly posits that CXCL16 functions primarily as a pro-inflammatory and pro-atherogenic factor in AS (115), earlier studies have indicated that mCXCL16 may exert a protective effect during the initial stages of AS by acting as a scavenger receptor (116). This observation implies that a moderate enhancement of mCXCL16 expression and accumulation, thereby augmenting its capacity to clear oxidized lipids and apoptotic cells, could contribute to plaque stabilization in early AS and decelerate disease progression. Nonetheless, the precise conditions under which mCXCL16 transitions from an anti-AS to a pro-AS role—encompassing factors such as expression levels, timing, and the cellular microenvironment—remain inadequately understood and warrant further investigation to substantiate these findings.

In the context of autoimmune diseases and chronic inflammation, the therapeutic objective is to mitigate the pathogenic effects associated with the CXCL16/CXCR6 axis. A pivotal strategy involves obstructing the recruitment of inflammatory cells to affected tissues, a process mediated by CXCR6, and diminishing the production of sCXCL16. Consequently, the regulation of the CXCL16/CXCR6 axis transcends a simplistic binary of ‘activation’ or ‘inhibition’; it necessitates a nuanced strategy of ‘condition-specific targeting.’ Intervention strategies should be tailored to the specific stage of the disease. During the early inflammatory phase, a moderate enhancement of CXCL16/CXCR6 may facilitate immune cell-mediated pathogen clearance. Conversely, in the chronic phase, it is imperative to suppress overactivation. Furthermore, precise regulation should focus on specific immune cell subsets expressing CXCR6, such as CXCR6^+^CD8^+^ T cells, Tregs, and NK cells, rather than adopting a universal approach. For instance, in EAE, the primary subset is CD4^+^ T cells, whereas in psoriasis, it is the Th17 cell subset. Moreover, localized interventions targeting the CXCL16/CXCR6 axis in specific tissues are advisable. In the case of rheumatoid arthritis, for example, targeting CXCL16/CXCR6 within the joints is preferable to systemic inhibition. In conclusion, by comprehensively understanding the disease stage, pathological characteristics, and target cell populations, selective and precise modulation of the CXCL16/CXCR6 axis can optimize therapeutic outcomes while minimizing adverse effects.

Current research on the CXCL16/CXCR6 axis encounters several significant challenges. Firstly, this axis demonstrates a ‘double-edged sword’ nature across various physiological and pathological contexts: it has the potential to both promote inflammation or cancer and engage in anti-inflammatory or anti-tumor responses. This inherent complexity poses substantial obstacles to the development of targeted therapies. Additionally, the temporal dynamics of the axis’s activity remain poorly understood, and there is a lack of precise definition regarding the ‘intervention window.’ Secondly, there are notable deficiencies in clinical research. At present, there is an absence of large-scale, prospective cohort studies to substantiate the utility of the CXCL16/CXCR6 axis as a reliable biomarker. Moreover, the animal models employed in preclinical studies exhibit differences in the expression and function of this axis compared to humans, thereby constraining the applicability of these findings to clinical settings. At the technical level, a standardized detection method that can accurately differentiate between sCXCL16 and mCXCL16 has yet to be established, thereby limiting its utility as a subtype biomarker. Drug development in this area encounters several challenges, including off-target effects, risks of immunosuppression due to systemic administration, and the potential for tumor cells to develop resistance through alternative pathways mediated by other chemokine receptors. Regarding fundamental mechanisms, several critical biological questions remain unanswered. For instance, the specific regulatory mechanism by which the ADAM protease family cleaves mCXCL16 is not yet fully understood, and the complexity of the downstream signaling network of CXCL16/CXCR6 has not been sufficiently investigated. These gaps in knowledge impede the development of precise regulatory strategies targeting this axis.

In conclusion, the CXCL16/CXCR6 axis holds the potential to transition from a promising basic research discovery to a clinically valuable tool, contingent upon the systematic resolution of the aforementioned challenges. It is important to recognize that, as a narrative review, this study is primarily limited by the subjective nature of literature selection, which may introduce selection bias. Additionally, the substantial heterogeneity among the included studies precludes conducting a quantitative synthesis.

## Data Availability

The original contributions presented in the study are included in the article/**Supplementary Material**. Further inquiries can be directed to the corresponding author.

## Glossary

mCXCL16: membrane-bound CXCL16
ADAM: disintegrin and metalloproteinase
Th: T helper cells
NSCLC: non-small cell lung cancer
AC: adenocarcinoma
CRC: colorectal cancer
PrEC: prostate epithelial cells
DLBCL: diffuse large B-cell lymphoma
MSCs: mesenchymal stem cells
CAFs: Cancer-associated fibroblasts
CTLs: cytotoxic T lymphocytes
GMZ: granzymes
DCs: dendritic cells
MS: multiple sclerosis
RA: rheumatoid arthritis
IL: interleukin
GM-CSF: granulocyte-macrophage colony stimulating factor
IgE: immunoglobulin E
ox-LDL: oxidized low-density lipoprotein
sCXCL16: soluble CXCL16
NK: natural killer cells
TME: tumor microenvironment
SCLC: small cell lung cancer
SCC: squamous cell carcinoma
PDAC: pancreatic ductal adenocarcinoma
GC: gastric cancer
TAMs: tumor-associated macrophages
MDSCs: myeloid-derived suppressor cells
TRM: tissue-resident memory T cells
PRF: perforin
MIBC: muscle-invasive bladder cancer
RCC: renal cell carcinoma
EAE: experimental autoimmune encephalomyelitis
TNF-α: tumor necrosis factor-alpha
IFN-γ: interferon-gamma
ROS: reactive oxygen species
LDL: low-density lipoprotein
AS: atherosclerosis
