# Supplementary material for: Advancements in understanding and treating CXCL16/CXCR6 in tumors and in inflammatory diseases: a narrative review
Source: Front Immunol. 2026 Jul 20;17:1802735. doi: 10.3389/fimmu.2026.1802735 (PMC13429434; doi:10.3389/fimmu.2026.1802735)
Supplement: Supplementary file 1 [file Table1.docx]

**Supplementary Materials**

**Supplementary Table S1. Details on CXCL16/CXCR6 in inflammatory diseases.**

| **Disease** | **Author** | **Year** | **Country** | **Species** | **Counts/n** | **CXCL16** | | | **CXCR6** | |
| --- | --- | --- | --- | --- | --- | --- | --- | --- | --- | --- |
|  |  |  |  |  |  | **Type** | **Sample Source** | **Detection method** | **Sample Source** | **Detection method** |
| Psoriasis | Schielke *et al.* | 2022 | Germany | Human | 49 | CXCL16 | Monocyte | NR | NR | NR |
| Psoriasis | Günther *et al*. | 2012 | Germany | Human | NR | sCXCL16  mCXCL16 | Serum | ELISA or IF | Serum or tissue | FC or IHC |
| Psoriasis | Steffen *et al.* | 2018 | Germany | Human | NR | sCXCL16  mCXCL16 | Serum or tissue | FC or ELISA or IF | Serum or tissue | IHC or ELISA |
| Psoriasis | Dong *et al.* | 2025 | China | Human or Mouse | 2/6 | CXCL16 | Serum or tissue | RNA-seq or IF | Serum or tissue | RNA-seq or IF |
| Psoriasis | Chen *et al.* | 2025 | China | Human or Mouse | 5/9 | CXCL16 | Tissue | FC or IF | Tissue | FC or IF |
| Pustular psoriasis | Lu *et al.* | 2023 | China | Human or Mouse | 5/26 | mCXCL16 | Serum or tissue | ELISA or IHC | Serum or tissue | ELISA or IHC |
| Covid-19 | Gregersen *et al.* | 2022 | Norway | Human | 414 | CXCL16 | Tissue | RNA-seq | Tissue | RNA-seq |
| Covid-19 | Smieszek *et al.* | 2022 | America | Human | 115 | sCXCL16 | Plasma or serum | ELISA | NR | NR |
| Sarcoidosis | Agostini *et al.* | 2005 | Italy | Human | 18 | CXCL16 | BAL or tissue | FC or IHC | BAL or tissue | FC or IHC or IF |
| Pulmonary sarcoidosis | Facco *et al.* | 2007 | Italy | Huamn | 33 | CXCL16 | BAL | Migratory assays | BAL or tissue | FC or IHC |
| Acute lung injury | Tu *et al.* | 2019 | China | Human | 20 | sCXCL16 | Serum | ELISA | NR | NR |
| Allergic asthma | Zou *et al.* | 2025 | China | Mouse | 6 | CXCL16 | NR | NR | Tissue | FC or RNA-seq |
| Asthma | Liu *et al.* | 2025a | China | Mouse | 6 | mCXCL16 | Tissue or BAL | IHC or FC or RNA-seq | NR | NR |
| Atherosclerosis | Meyer Dos Santos, S., *et al.* | 2015 | Germany | Human | NR | mCXCL16 | Tissue | IHC | Platelet | ICC |
| Atherosclerosis | Linke, B., *et al.* | 2019 | Germany | Human | NR | mCXCL16 | Tissue; HUVECs | IHC or ICC or MELC | Serum or tissue | IHC or ICC or MELC |
| Atherosclerosis | Aslanian *et al.* | 2006 | American | Human or Mouse | 10/11 | sCXCL16 mCXCL16 | Serum or tissue | ELISA/IHC/FC | Tissue or cell | qPCR or FC |
| Atherosclerosis | Lehrke, M., *et al.* | 2007 | American | Human | NR | sCXCL16 mCXCL16 | Serum or tissue | ELISA or IHC | NR | NR |
| Atherosclerosis | Zivković *et al.* | 2015 | Serbia | Human | 450 | sCXCL16 mCXCL16 | Serum or tissue | ELISA or IHC | Tissue | qPCR |
| Atherosclerosis | Hu, Z.B., *et al.* | 2016 | China | Human | NR | sCXCL16 mCXCL16 | Serum or tissue | ELISA or IHC | NR | NR |
| Atherosclerosis | Wuttge, D.M.,*et al.* | 2004 | Sweden | Human | NR | mCXCL16 | tissue | IHC or qPCR | Tissue | qPCR or IHC |
| Atherosclerosis | Liu, Y., *et al.* | 2025 | China | Human | NR | sCXCL16 mCXCL16 | Serum or tissue | ELISA or IHC | NR | NR |
| Rheumatoid Arthritis | Nanki, T., *et al.* | 2005 | Japan | Human or Mouse | NR/8 | mCXCL16 | Tissue(Mouse) | IHC or IF | Tissue(Human) | IHC |
| Rheumatoid Arthritis | Zhang, X., *et al.* | 2017 | China | Human | 15 | mCXCL16 | Tissue | Western blot or ELISA | Tissue | WB |
| Rheumatoid Arthritis | Turgunova, L.G., *et al.* | 2021 | American | Human | NR | sCXCL16 mCXCL16 | Serum or tissue | ELISA or IHC | NR | NR |
| Juvenile Idiopathic Arthritis | Martini, G., *et al.* | 2008 | Italy | Human | NR | NR | NR | NR | Tissue | IHC |
| Arthritis-related inflammation | Li, C., *et al.* | 2012 | China | Human | NR | mCXCL16 | Tissue | Western blot or IHC | NR | NR |
| Rheumatoid Arthritis | Li, C.H., *et al.* | 2016 | China | Human | NR | mCXCL16 | Tissue | Western blot or IHC | Tissue | WB or IHC |

NR: Not Reported.

IHC: Immunohistochemistry

FC: Flow Cytometry

BAL: Bronchoalveolar Lavage fluid

RNA-Seq: RNA Sequencing

WB: Western Blot

ICC: Immunocytochemistry

MELC: Micellar Electrokinetic Chromatography
